# Development and Psychometric Properties of a Self-Efficacy Scale for Healthy Meals in a Diverse Sample of Adults

**DOI:** 10.21203/rs.3.rs-6763050/v1

**Published:** 2025-06-05

**Authors:** Lorena Macias-Navarro, Natalia I. Heredia, Timothy J. Walker, Jennifer S. Massa, Miranda A. Moore, Samiha Momin, Deanna M. Hoelscher

**Affiliations:** The University of Texas Health Science Center at Houston (UTHealth Houston); The University of Texas Health Science Center at Houston (UTHealth Houston); The University of Texas Health Science Center at Houston (UTHealth Houston); Harvard T.H. Chan School of Public Health; Emory University; The University of Texas Health Science Center at Houston (UTHealth Houston); The University of Texas Health Science Center at Houston (UTHealth Houston)

**Keywords:** self-efficacy, confirmatory factor analysis, scale development, psychometric validation, healthy meals, cooking barriers

## Abstract

**Background:**

Many U.S. adults do not meet recommended dietary guidelines, partly due to low self-efficacy (SE) in overcoming barriers to preparing healthy meals. Existing SE measures are often lengthy, lack psychometric testing, and do not assess these barriers. To address this gap, we developed the Self-Efficacy Scale for Healthy Meals and examined its structural validity and reliability.

**Methods:**

The SE Scale for Healthy Meals was constructed following established scale development guidelines and refined through expert reviews, reducing an initial pool of 40 items to 11. We conducted confirmatory factor analysis (CFA) using a diverse sample of 139 adults, recruited both online and from a lifestyle medicine trial. CFA models tested one-factor (“Self-Efficacy”) and two factor (“Situational Barriers” and “Resource Barriers”) structures using robust maximum likelihood estimation. Construct validity was assessed through model fit indices, factor loadings, and inter-item correlations, while internal consistency reliability was evaluated using Cronbach’s alpha and Raykov’s rho.

**Results:**

The two-factor model demonstrated superior fit (RMSEA = 0.042; SRMR 0.052; CFI = 0.963; TLI = 0.952) compared to the one-factor solution, confirmed by a significant Satorra-Bentler scaled chi-square difference test (ΔSB c^2^ = 21.93, p < 0.001). Internal consistency reliability was acceptable to good (Cronbach’s a and Raykov’s r > 0.70).

**Conclusions:**

The SE Scale for Healthy Meals has adequate psychometric properties, including good reliability and structural validity, when conceptualized as a two-factor measure. This tool is useful for designing and evaluating interventions to enhance cooking SE and promote healthy dietary behaviors.

## Background

Despite widespread public health efforts to promote adherence to the Dietary Guidelines for Americans (DGA), overall diet quality in the United States remains suboptimal (Shams-White et al., 2023). According to the most recent Healthy Eating Index (HEI-2020), a tool that evaluates adherence to the DGA, Americans aged 2 and older have and average score of approximately 58 out of 100, far below the ≥80 benchmark (Shams-White et al., 2023). Low HEI scores have been consistently associated with higher odds of overweight and obesity (Reedy, Jill et al., 2014) and increase the risk for chronic conditions, including type 2 diabetes and heart disease, contributing to premature morbidity and mortality (Fanelli et al., 2020).

Growing evidence supports that an individual’s ability and confidence to cook nutritious meals at home is associated with improvements in diet quality (Lo et al., 2019). Adults who report higher cooking self-efficacy tend to have higher HEI scores compared to those with lower self-efficacy (Wolfson et al., 2020). Additionally, multicomponent programs that offer a combination of food delivery, kitchen skill-building, and hands-on cooking classes showed improvement in participants’ cooking confidence and dietary behaviors (Mendez et al., 2021; Ylitalo et al., 2023). However, most existing research primarily emphasizes enhancing cooking skills and confidence without fully exploring the psychological and practical barriers individuals face when applying these skills in daily life (Lo et al., 2019; Mendez et al., 2021; Ylitalo et al., 2023). Addressing this gap requires interventions specifically designed to strengthen psychosocial constructs, such as self-efficacy, to effectively help individuals overcome barriers to adopting and sustaining healthy eating behaviors.

Self-efficacy (SE), the core construct in the Social Cognitive Theory (SCT), is defined as a person’s confidence in their ability to perform and overcome barriers related to a specific behavior (Bandura, 1986). SE is strongly associated with behavior change, including nutrition-related behaviors, making it a key construct to inform and design public health interventions to improve dietary behaviors (Lavelle et al., 2020; Reedy, J. et al., 2018; Wilson et al., 2016).

A critical barrier to improving dietary behaviors is an individual’s lack of SE (confidence) to overcome barriers to prepare and cook healthy meals (Mcgowan et al., 2017). With the widespread availability of highly processed foods and ready-to-eat affordable meals, preparing healthy meals at home has become less of a priority and the ability of individuals to overcome barriers to prepare healthy meals at home has decreased, leading to poor dietary behaviors (Anderson et al., 2007; Mcgowan et al., 2017). Evidence suggests that there is an association between higher confidence in the abilities to prepare and cook healthy meals with an improved overall dietary intake (Mcgowan et al., 2017; Thorpe et al., 2014). However, individuals often face multiple barriers, such as lack of cooking skills, time constraints, low motivation, stress, or limited access to healthy ingredients that limit their ability to prepare healthy meals (Ashton et al., 2017; Bross et al., 2022; Cheong et al., 2022; Garcia et al., 2021). Overcoming these barriers thus requires both practical cooking skills and confidence to consistently engage in healthy meal preparation despite various challenges (Mcgowan et al., 2017).

Existing studies assessing barriers to healthy eating are mostly qualitative studies (Cheong et al., 2022; Garcia et al., 2021) and the few quantitative studies available often lack an examination of the structural validity and reliability of SE measures (Ashton et al., 2017; Bross et al., 2022). Some of the existing quantitative measures are not pragmatic (> 50 items per questionnaire) and may contribute to survey fatigue and respondent burden. While previous research has examined SE to adhere to healthy diets and to cook and prepare meals (Anderson et al., 2007; Greiner et al., 2018; Sebastian et al., 2021; Vilaro et al., 2016), there are no known measures that assess cooking SE in the context of overcoming barriers.

To address this gap, we developed an 11-item questionnaire to assess SE in overcoming barriers to preparing and consuming healthy meals at home. This measure was designed as part of the Teaching Kitchen Multisite Trial (TKMT), led by the Harvard T.H. Chan School of Public Health in collaboration with UTHealth Houston School of Public Health and other participating research institutions (Massa et al., 2025). The TKMT is a year-long, lifestyle medicine randomized controlled trial of adults who are overweight or with class I-II obesity (Body Mass Index 25–39.9 kg/m2). The TKMT aims to improve health and wellness status by teaching culinary skills and nutrition education, among other lifestyle medicine components (Massa et al., 2025).

The purpose of this manuscript is to describe the development process of the Self-Efficacy Scale for Healthy Meals questionnaire and evaluate its structural validity and reliability. Using data from a diverse sample, including adults recruited online and adults participating in the TKMT, we conducted a confirmatory factor analysis to compare the model fit of one-factor and two-factor solutions.

## Methods

### Measurement Development

The development of the SE Scale for Healthy Meals was informed by DeVellis’s scale development process (DeVellis & Thorpe, 2021). First, the theoretical construct of self-efficacy, specifically an individual’s confidence in overcoming barriers to preparing and consuming healthy meals at home, was defined based on Bandura’s theoretical model (Bandura, 1986) and previous research on cooking and self-efficacy (Guntzviller et al., 2017; Jomori et al., 2022).

Second, an initial pool of items was created based on the review of nutrition related research articles from two main databases, PubMed and The Cochrane Library, using the following search terms: [“self-efficacy” OR “cooking self-efficacy” OR “cooking barriers”] AND [“healthy eating” OR “meals at home” OR “home cooking”] AND [“adults”]. As a result of this review, cross-sectional survey (Ashton et al., 2017), meta-synthesis (Cheong et al., 2022), and qualitative studies on the subject were found (Beck et al., 2019; Bross et al., 2022; Garcia et al., 2021; Vélez-Toral et al., 2020). These sources revealed similar SE scales for cooking which were used to identify recurring themes, such as food prices, availability, and cooking confidence, which informed the initial 40-item pool. The two sub-themes that emerged from this research were *situational barriers*, such as time constraints and stress, and *resource barriers*, such as limited access to affordable and nutritious food.

Third, based on Bandura’s conceptualization of *self-efficacy* (Bandura, 1986) and informed by previous research assessing cooking self-efficacy (Condrasky, M. D. & Griffin, 2007; Condrasky, Margaret D. et al., 2011; Guntzviller et al., 2017; Jomori et al., 2022; Kedem et al., 2014), we used a unipolar, 5-point Likert-type scale, where 1 indicates “not at all confident” and 5 indicates “extremely confident” (Appendix 1). Labelling all five points, rather than only the anchors, minimized ambiguity and improved clarity.

Furthermore, we included a neutral option since we were interested in understanding if respondents’ self-confidence is “neutral” regarding certain statements.

Finally, the draft scale underwent expert evaluation using the Davis technique (Davis, 1992). Ten experts affiliated with the TMKT, including six faculty members working in the field of nutrition and behavioral sciences, two culinary medicine instructors, and two doctoral students in health promotion and behavioral sciences and community nutrition, reviewed the items for comprehensibility and relevance. Subsequently, the TKMT experts ranked the items according to relevance and selected 11 final items to be included as part of the self-efficacy scale. Items were selected to capture a range of potential barriers while ensuring relevance to the *self-efficacy* construct, as well as the two sub-themes of *situational* and *resource barriers*.

### Measure

The SE for Healthy Meals is an 11-item unipolar self-reported measure to assess confidence in preparing healthy meals at home under various challenging conditions. The stem question reads, “*How confident are you that you can cook a healthy meal at home on a regular basis when…*” followed by 11 barrier-specific scenarios. These scenarios include *elevated food prices, limited time, feeling stressed, convenience of fast food, lack of nutrition knowledge, limited access to recipes or ingredients, difficulty meeting family preferences, burden of cleaning up after cooking, and concerns about food waste* (Table 1). Response options include 1 = not at all confident, 2 = not very confident, 3 = neutral, 4 = confident, and 5 = extremely confident. Higher scores indicate more self-efficacy to overcome barriers to cooking and consuming healthy meals.

### Data Collection and Study Participants

Data for this study were collected from two different sample sources that were pooled or analyses. The first sample of data (general population) was collected from an electronic questionnaire distributed to adults aged 18 and older who consented to the study by choosing to complete the questionnaire. Exclusion criteria were the inability to write or read in English. We distributed the questionnaire via email and social media posts (e.g., LinkedIn and X) using an online survey program (Qualtrics) in the spring and summer of 2024. The survey took approximately 10 minutes or less to finish. The questionnaire included demographic questions (gender, age, race/ethnicity, and education level), and the 11 items of the SE Scale for Healthy Meals. Participants had the option to mark “prefer not to respond” on all questions.

The second sample of data was from participants in the Teaching Kitchen Multisite Trial (TMKT), at the UTHealth Houston School of Public Health research site. The inclusion criteria included adults aged 21 to 70 years, with a diagnosis of overweight, class I or II obesity, and at least one cardiometabolic abnormality (e.g., fasting glucose, liver function, fasting lipids including triglycerides) (Massa et al., 2025). Most of the study participants were recruited from institutions within the Texas Medical Center, and from local television interviews and social media posts associated with UTHealth Houston (Massa et al., 2025). The SE Scale for Healthy Meals was administered during baseline assessments for participants randomized into the intervention and control groups during the spring of 2024.

This study was approved by the Institutional Review Board of the University of Texas Health Science Center at Houston, HSC-SPH-22–0924 for the TKMT intervention and HSC-SPH-24–0066 to distribute the SE questionnaire to a wider population, using an electronic consent, embedded into the survey.

### Analysis

All analyses and reporting adhered to the STROBE (Strengthening the Reporting of Observational Studies in Epidemiology) checklist for cross-sectional studies. These analyses were conducted on the 11 items of the SE Scale for Healthy Meals. All items were rated on a 5-point Likert scale ranging from 1 = not at all confident to 5 = extremely confident, with higher scores indicating greater self-efficacy. We used Stata version 16.1 (StataCorp LLC, College Station, TX) and Mplus version 8.6 for statistical analyses. Stata was used to calculate descriptive statistics and conduct initial reliability analyses, while both Stata and Mplus were used for the confirmatory factor analyses (CFAs) comparing one-factor and two-factor structures.

We first examined descriptive statistics for each item including means, standard deviations, skewness, and kurtosis, to evaluate item distribution and assess univariate normality. The dataset was also examined for missing data; participants with > 10% missing data were excluded using listwise deletion, following recommended practices (Dong & Peng, 2013).

Informed by behavioral theory literature and prior research on nutrition-related self-efficacy measurements (Condrasky, M. D. & Griffin, 2007; Condrasky, Margaret D. et al., 2011; Guntzviller et al., 2017; Jomori et al., 2022; Kedem et al., 2014), we specified two CFA models using maximum likelihood estimation with robust standard error (MLR) in Mplus to account for non-normality. Specifically, we estimated a one-factor model, with all items loading on a single latent construct representing “Self-Efficacy” and a two-factor model, with one latent factor representing “Situational Barriers” and a second factor reflecting “Resource Barriers.”

To evaluate construct validity, we examined inter-item correlations to assess discriminant validity (correlations < 0.80) and conducted confirmatory factor analyses (CFAs) to test the factorial structure of the scale (Brown, 2015). Model fit was evaluated using multiple indices: the chi-square test, Comparative Fit Index (CFI), Tucker-Lewis Index (TLI), Root Mean Square Error of Square Residual (SRMR). Following conventional guidelines, model fit was considered acceptable with CFI and TLI values ≥ 0.90, RMSEA ≤ 0.08, and SRMR < 0.08 (Byrne, 2013). Factor loadings were examined for each model to evaluate the strength of item-factor relationships.

To compare the nested CFA models under MLR, we used the Satorra-Bentler scaled chi-square difference test (Satorra & Bentler, 2001). Given that traditional chi-square difference testing is not valid with robust estimation, we applied the corrected formula in Excel using model-specific chi-square values, degrees of freedom, and scaling correction factors from Mplus output. This method allowed us to test whether increased model complexity significantly improved fit while accounting for non-normality (Satorra & Bentler, 2001).

For internal consistency, we first computed Cronbach’s alpha (> 0.70 = acceptable values). We also calculated Raykov’s rho, a CFA-based reliability estimate, that accounts for unequal factor loadings and uses both standardized loadings and residual variances (Raykov, 2004). Unlike Cronbach’s alpha (a), which assumes tau-equivalence, Raykov’s rho (r) provides a more flexible and theoretically grounded estimate of scale reliability (Brown, 2015; Raykov, 2001). We also calculated 95% confidence intervals for Raykov’s rho using the delta method (Brown, 2015; Raykov, 2001).

## Results

Given the small sample sizes (general population = 147; TKC intervention participants = 31), we pooled data from both groups for analysis (total n = 178). Of these, 32 individuals accessed the survey link but did not initiate the survey, and an additional 7 participants provided incomplete responses with more than 70% missing survey data. Thus, a total of 39 surveys were not included in the analysis because they were blank or had substantial missing data. Data from 139 complete surveys, including 108 respondents from the general population and 31 respondents (baseline assessments for control and intervention groups) currently participating in the TKC lifestyle medicine intervention program, were included in the final analysis.

The sample was predominately female (> 80%) and Hispanic (61%). Moreover, participants were highly educated, with more than 50% holding college degrees and postgraduate degrees. This high level of education reflects the recruitment of most participants from academic/research institutions within the Texas Medical Center. Additionally, the sample was also racially and ethnically diverse (Table 2).

Among the 11 self-efficacy items, Item 4, which asked about confidence to cook a healthy meal “*when it’s easier to buy fast food*,” showed the lowest correlations with the other items on the scale ranging from 0.07 to 0.24 (Table 3). This suggests that the fast-food barrier may be perceived as a more external barrier, distinct from resource or situational barriers. Moreover, the lowest mean self-efficacy scores were observed from Item 2 (“*when you don’t have enough time to cook*”; μ = 2.98, SD = 1.23) and Item 3 (“*when you feel stressed or overwhelmed*”; μ = 2.92, SD = 1.14), suggesting that these scenarios present the greatest challenges for preparing healthy meals. In contrast, the highest self-efficacy mean was found for Item 10 (“*when you have to clean up after cooking*”; μ = 3.82, SD = 1.10), suggesting that this task is perceived as least difficult to overcome, compared to the other scenarios (Table 4).

### Factor Structure and Construct Validity

**Figure 1:**
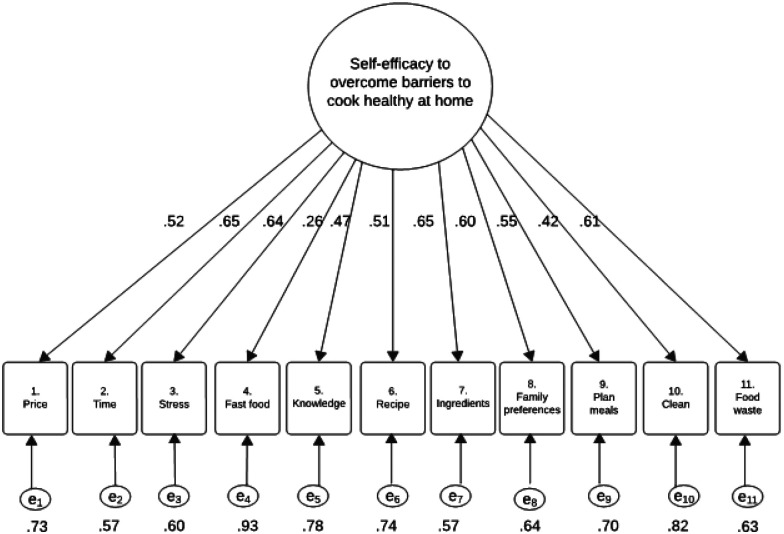
Visual Representation of a One Factor Model for the 11 items of the Self-Efficacy Scale for Healthy Meals

The initial CFA examined a one-factor solution in which all 11 items of the self-efficacy scale loaded onto a single latent factor (Fig. 1). The standardized factor loadings ranged from 0.26 to 0.65, where most items demonstrated moderate loadings (≥ 0.50). The highest loading was observed for the Item 7 (“*when you have limited ingredients at home*”), followed by Item 2 (“*when you do not have enough time to prepare healthy meals*”), and Item 3 (“*when you are feeling stressed*”), suggesting these are central barriers in maintaining healthy meal practices. On the other hand, the lowest loading was observed for Item 4 (“*when it is easier to buy fast food than cook at home*”), indicating a weak relation with the general factor.

### Self-Efficacy Scale for Healthy Meals

**Figure 2:**
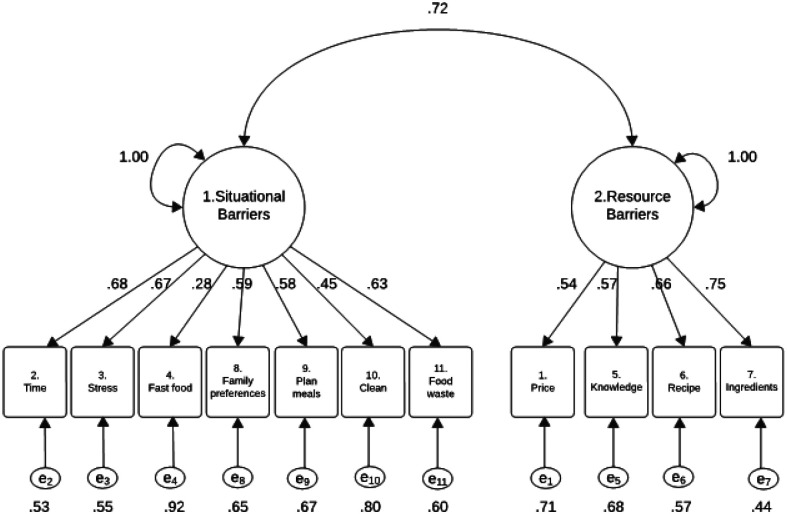
Visual Representation of a Two Factor Model for the 11 items of the Self-Efficacy Scale for Healthy Meals

In the two-factor CFA model, standardized factor loadings ranged from 0.279 to 0.746 (Fig. 2), indicating varying levels of association between items and their respective latent constructs. Most items demonstrated moderate to strong loadings (≥ 0.50), consistent with recommended thresholds (Brown, 2015). For Factor 1 (Situational Barriers), higher loadings were related to Item 2 (“You do not have enough time to prepare healthy meals”) and Item 8 (“*when it is difficult to cater to your family’s preferences*”), with the exception of Item 4 (“*when it is easier to buy fast food than cook at home*”), which had a notably lower loading and may reflect a conceptually distinct barrier. Factor 2 (Resource Barriers) showed strong and consistent loadings across all four items, with Item 7 (“*when you have limited ingredients at home*”) loading the highest. These results support the hypothesized two-factor structure and suggest that most items contributed meaningfully to their respective constructs, except for the low-loading Item 4, which may require refinement or further examination in future scale development.

### Self-Efficacy Scale for Healthy Meals

#### Dimensionality of the scale: Two-facto model versus one-factor model

Results from the one and two-factor models are displayed on Table 5. The one-factor model had evidence of poor model fit (χ2(df = 44) = 76.165, p < 0.001; RMSEA = 0.073; SRMR 0.064; CFI = 0.887; TLI = 0.859), and the two-factor model had good model fit (χ2(df = 43) = 53.638, p > 0.05; RMSEA = 0.042; SRMR 0.052; CFI = 0.963; TLI = 0.952). Model comparison using the Satorra-Bentler scaled chi-square difference test revealed the two-factor model better fit the data (ΔSB c^2^ = 21.93, p < 0.001). Thus, these results support the selection of the two-factor structure as the best-fitting model.

#### Internal Consistency Reliability

To assess internal consistency reliability, we calculated both Cronbach’s alpha (a) and Raykov’s rho (r). For the one-factor model, the scale demonstrated high reliability, with a = 0.81 and r (0.816, 95% CI = [0.772–0.860]). For the two-factor model, the Situational Barriers subscale showed acceptable reliability (a = 0.76; r (0.761, 95% CI = [0.706–0.816]), and the Resource Barriers subscale also demonstrated adequate internal consistency (a = 0.72; r (0.721, 95% CI = [0.648–0.794]). These findings suggest that the scale and its subscales have acceptable to good internal consistency across different model specifications (Table 6).

## Discussion

This study developed and examined the validity and reliability of an 11-item SE Scale for Healthy Meals. Our findings suggest that the developed measure has adequate psychometric properties, including good reliability and structural validity. The confirmatory factor analyses favored a two-factor solution, with two distinct subscales: “*Situation Barriers*” and “*Resource Barriers*.” Both subscales demonstrated good internal consistency and acceptable fit indices. This differentiation supports that individuals’ self-efficacy for preparing and consuming healthy meals at home varies based on context-dependent or systemic barriers.

The theorical framework for this distinction is grounded in Bandura’s SCT, which indicates that self-efficacy belief is sensitive to the individuals’ perception of barriers and their context (Bandura, 1986). Situational barriers, such as time constraints and stress, are often temporary and can be mitigated with targeted interventions to manage stress or meal planning (Anderson et al., 2007; Greiner et al., 2018; Sebastian et al., 2021; Vilaro et al., 2016). In contrast, resource barriers including limited access to ingredients or lack of culinary knowledge, reflect systemic or knowledge-based obstacles that may require the provision of materials or educational programs for skill-building (Anderson et al., 2007). By conceptually differentiating these two constructs, we provide a framework for designing and evaluating more precise interventions to enhance healthy cooking practices.

One notable finding was that item 4 (“*when it’s easier to buy fast food than cook at home*”) consistently showed low factor loadings in both models. This may suggest that respondents do not view fast food as a barrier to cooking at home, possibly because it has become a normalized and routine part of the food environment (Dunn et al., 2014). Moreover, the ubiquity and convenience of fast food can diminish its perceived role as a barrier, even when it competes with healthier behaviors (Potvin Kent et al., 2024). Another possible explanation for low factor loadings may be that the item’s wording does not effectively capture the intended construct. While retaining this item allowed us to analyze the complete set of 11 items, future research may consider revising the wording or removing this item to improve the overall validity of the measure.

Previous SE measures have focused on global confidence in performing specific cooking skills (e.g., sautéing, grilling), rather than capturing the nuanced barriers individuals face in their daily lives when preparing healthy meals. (Garcia et al., 2021; Vélez-Toral et al., 2020)The measure developed in this study is uniquely designed to assess situational and resource-related barriers to engage in healthy home cooking. This is particularly important to inform the design of effective culinary medicine programs aimed at improving confidence in cooking and preparing healthy meals at home.

### Limitations

This study has several limitations. First, the scale was tested using a sample of highly educated, diverse adults, which limits the generalizability of findings to a wider population. Second, the study did not account for social desirability bias, leaving uncertainty regarding its potential impact on responses. Third, the relatively small sample size reduces statistical power and may lead to biased parameter estimates, limiting the confidence in the replicability of the findings. Fourth, the study focused solely on structural validity and internal consistency, indicating that further testing to assess other aspects of validity and reliability are needed. Future research should test this scale with larger, more diverse samples of adults.

## Conclusions

In conclusion, the SE Scale for Healthy Meals demonstrates promising psychometric properties with a two-factor structure that differentiates situational and resource barriers. Despite limitations including a small, highly educated sample, the measure offers a novel, targeted approach for assessing self-efficacy in interventions involving healthy home cooking skills. A deeper understanding of these factors can guide the development of effective culinary medicine programs. Valid and reliable measures are crucial to better inform our understanding of individual’s confidence in preparing meals at home to achieve and maintain a healthy diet and improving overall health.

## Figures and Tables

**Table 1:** Self-Efficacy for Healthy Meals Scale

How confident are you that you can cook a healthy meal at home on a regular basis WHEN:	
1	The price of healthy food goes up.
2	You do not have enough time to prepare healthy meals
3	You are feeling stressed
4	It is easier to buy fast food than cook at home
5	You don’t know what food is healthy.
6	You don’t have a recipe to follow.
7	You have limited ingredients at home.
8	It is difficult to cater to your family’s preferences
9	You haven’t planned your meals for the week
10	You have to clean up after cooking
11	You are concerned about purchasing foods that will go to waste

Response options: 1 = not at all confident, 2 = not very confident, 3 = neutral, 4 = confident, and 5 = extremely confident.

**Table 2:** Descriptive Statistics of the Pooled Study Sample (N = 139)

Variables	Sample 1 (n = 108)	Sample 2 (n = 31)
	n (%)	n (%)
**Gender**		
Male	19 (18)	5 (16)
Female	87 (80)	25 (81)
Prefer not to respond	2 (2)	1 (3)
**Race**		
White or Caucasian	49 (45)	19 (61)
Black or African American	37 (34)	3 (10)
Asian or Asian American	13 (12)	4 (13)
American Indian & Other	5 (5)	3 (10)
Prefer not to respond	4 (4)	2 (6)
**Ethnicity**		
Hispanic	66 (61)	19 (61)
Not Hispanic	23 (21)	11 (36)
Prefer not to respond	19 (18)	1 (3)
**Education**		
Less than high school, high school, and some college but no degree	17 (15)	0
College graduate	30 (28)	8 (26)
Postgraduate	60 (56)	23 (74)
Prefer not to respond	1 (1)	0
	Mean (SD)	Mean (SD)
**Age**	37.5 (12.73)	38 (10.86)

Note. SD = standard deviation.

**Table 3:** Correlations matrix for the SE Scale

Measure/Items	1	2	3	4	5	6	7	8	9	10	11
1.Price	1										
2.Time	0.31	1									
3.Stress	0.31	0.54	1								
4.Fast food	0.15	0.19	0.23	1							
5.Knowledge	0.33	0.29	0.28	0.13	1						
6.Recipe	0.32	0.31	0.27	0.07	0.47	1					
7.Ingredients	0.37	0.39	0.36	0.15	0.39	0.55	1				
8.Family’s preferences	0.28	0.36	0.36	0.24	0.37	0.23	0.42	1			
9.Meal plan	0.23	0.45	0.34	0.19	0.16	0.32	0.37	0.36	1		
10.Cleaning	0.26	0.31	0.23	0.17	0.11	0.14	0.18	0.36	0.34	1	
11.Food waste	0.33	0.4	0.5	0.17	0.26	0.24	0.26	0.41	0.36	0.44	1

**Table 4:** Descriptive statistics for the SE Scale for Healthy Meals

Items	Mean	SD	Skewness	Kurtosis
**1.Price**	3.7	1.08	−0.56	2.42
**2.Time**	2.98	1.23	−0.06	1.92
**3.Stress**	2.92	1.14	0.17	1.98
**4.Fast food**	3.35	1.19	−0.41	2.12
**5.Knowledge**	3.3	1.31	−0.29	1.94
**6.Recipe**	3.4	1.27	−0.47	2.18
**7.Ingredients**	3.19	1.15	−0.04	2.02
**8.Family’s preferences**	3.19	1.15	−0.13	2.17
**9.Meal plan**	3.15	1.17	−0.04	2.01
**10.Cleaning**	3.82	1.1	−0.7	2.57
**11.Food waste**	3.5	1.17	−0.3	2.06

SD = standard deviation

**Table 5:** Global Fit Indices for Multiple Models

	c^2^ (df)	CFI	TLI	RMSEA	RMSEA 90% CI	SRMR	Model Comparision	ΔSB c^2^
Model 1	76.165 (44) *	0.887	0.859	0.073	0.044 – 0.099	0.064		
Model 2	53.638 (43)	0.963	0.952	0.042	0.00 – 0.075	0.052	1 vs. 2	21.926*

Note. Model 1 = One factor solution; Model 2 = Two factor solution. df = Degrees of freedom; CFI = Comparative fit index, TLI = Tucker-Lewis Index; RMSEA = Root mean square error of approximation; CI = confidence interval; SRMR = Standardized root mean square residual. ΔSB c^2^ = Satorra–Bentler scaled chi-square difference test;

* p < 0.001.

**Table 6:** Point Estimation of the SE Scale Reliability

	Cronbach’s alpha	Raykov’s rho (95% CI)
**One factor (Model 1)**		
11 items	0.8129	0.816 (0.772–0.860)
**Two factors (Model 2)**		
Situational barriers (Items 2,3,5,8,9,10, 11)	0.7563	0.761 (0.706–0.816)
Resource barriers (Items 1,5,6,7)	0.7213	0.721 (0.648–0.794)

## Data Availability

The datasets used and/or analyzed during the current study are available from the corresponding author on reasonable request.
